# Molecular Characterization of the Measles Virus Genotypes in JiLin Province, China

**DOI:** 10.1371/journal.pone.0046011

**Published:** 2012-10-08

**Authors:** Chengguo Wei, Jingwei Shi, Bin Liu, Yue Shi, Jingtong Zheng, Guangyu Xu, Jinshu Ma, Guoqing Wang, Fan Li

**Affiliations:** 1 Department of Pathogeny Biology, Norman Bethune Medical College of Jilin University, Changchun, Jilin, China; 2 Key Laboratory of Zoonosis Research, Ministry of Education, Jilin University,, Changchun, Jilin, China; 3 The First Bethune Hospital of Jilin University, Changchun, Jilin, China; Centro Nacional de Microbiología - Instituto de Salud Carlos III, Spain

## Abstract

Measles remains a severe global health threat, and nearly 30 million new cases are reported annually. Although many studies have analyzed measles viruses (MV) at the epidemiologic and phylogenetic levels, no study has yet to integrate these two types of data. To this end, we isolated 16 wild-type MV strains China's Jilin province. The MV genotype H1 was the most prevalent strain. After sequencing the nucleoprotein (N) genes of these strains, a maximum clade credibility tree was constructed by the Bayesian Markov Chain Monte Carlo method using 450 MV strains from GenBank with epidemiological information. The MV N gene evolution rate was 1.127E-3. Analysis of the time of the most recent common ancestor (TMRCA) for genotypes A/B/C/G/H revealed that genotypes D and B had the largest and smallest TMRCA (45.86 and 26.63, respectively). The highest level of genetic diversity for the MV N gene occurred around the year 2000. Here in this study, we uncovered the MV genotypes circulating in China's Jilin Province and estimated the epidemiologic and phylogenetic relationship for the six different genotypes of MV.

## Introduction

Measles virus (MV) is a negative-strand RNA paramyxovirus, and causes an acute infectious disease and a chronic neurological disorder, subacute sclerosing panencephalitis (SSPE) [1], [2]. Despite the development of vaccines against MV and widespread vaccination campaigns, measles remains one of the most contagious diseases and a leading cause of child death worldwide [3]. More than 30 million new cases are reported annually, the majority of which are in children. A staggering number of measles-related deaths (up to 95%) occur in developing countries [4], due to their limited healthcare resources China is no exception to this rule, despite its recent advances in economic standing. In Jilin Province, home to over 27 million individuals, measles remains the most deadly of all childhood rash/fever illnesses [5]. In 2009, the incidence of measles was still remarkably high, about 10 incidences/100,000 population [5].

The World Health Organization (WHO) has recognized eight clades of MV (designated A–H) that encompass 23 genotypes [6]. Some of the genotypes in these clades represent the prominent sporadic and outbreak-associated infections that have occurred across the globe over the past four decades: A, sporadic infections [7]; B, Africa-related genotypes [8]; D, genotypes implicated in outbreaks in the United States, Pakistan, India [9], and Japan [10]; and H1, the China-related genotype [11]. Thus far, the G genotype appears to be inactive [12]. At this point, a study to determine the MV genotype distribution of currently circulating MV strains will provide crucial insights into the epidemiologic and phylogenetic features of the disease in Jilin Province, thereby providing necessary information to create more effective vaccination strategies and transmission prevention.

The MV genome-encoded nucleoprotein (N) is critically involved in viral genome transcription and replication, frequently used for genotyping and phylogenetic analysis [13]. However, no study to date has reported on the relationship between the genetic characteristics of the various MV genotypes and their epidemiologic behavior. Such data would help to identify the source of virus for a particular region or population [14]. While next-generation sequencing technologies (such as Illumina's Solexa, 454's FLX, or Applied Biosystem's SOLiD) allow for rapid and in-depth genomic analysis, the newly developed bioinformatic methods (such as Bayesian [15]) allow for meaningful analysis of the evolutionary and epidemiological behavior of the sequencing data.The classic methods used to study system evolution include distance, maximum parsimony (MP), and maximum likelihood (ML) [16]. The newly proposed Bayesian method [15] not only retains the basic principle of the ML method but also introduces the Markov chain Monte Carlo method, which greatly reduces the calculation time. In addition, the Bayesian method uses the posterior probability to visually represent the phylogenetic relationships, thereby eliminating the need for bootstrapping.

In the current study, we isolated 16 measles viruses from 105 patient samples from five different cities in Jilin Province between 2005 and 2006. The N gene was sequenced from each MV sample and used to characterize the strain and perform epigenetic and phylogenetic analysis with the worldwide pool of MV strains published in GenBank (http://www.ncbi.nlm.nih.gov/nucleotide). Bayesian analyses were performed to construct a maximum clade credibility (MCC) tree and estimate the time of the most recent common ancestor (TMRCA) for all isolated and downloaded strains. Finally, Bayesian skyline plot analyses was used to reconstruct the past population history of MV by measuring the dynamics of N gene genetic diversity over time.

## Results and Discussion

### Phylogenetic analysis

**Figure 1:**
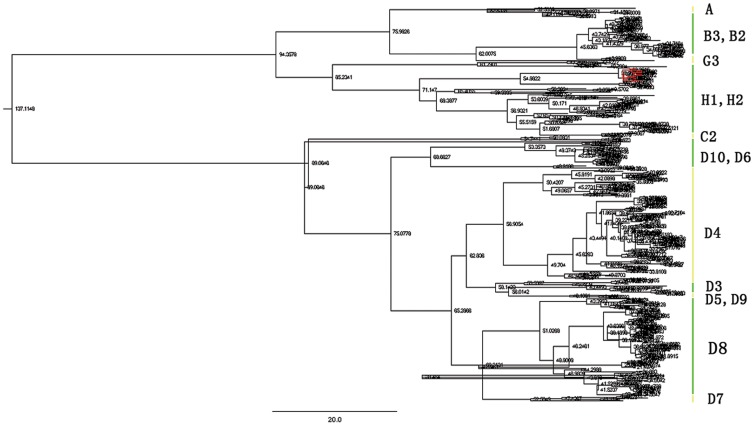
The maximum clade credibility tree was estimated by Bayesian analysis of nucleoprotein gene sequences with ∼450 bp of 466 measles virus strains. The posterior probabilities of the key nodes are depicted above the respective nodes. Samples isolated by our laboratory (n = 16, red lines) were analyzed with the other worldwide strains that were downloaded form GenBank. The green and yellow vertical lines indicate different genotypes. The original file containing more details is available as File S2, which can be opened with FigTree v1.3.1 software to be amplified for more details.

Of the 82 throat swabs and 23 urine samples from suspected MV patients, 16 were successfully isolated with Vero/SLAM cells and tested positive for the MV N gene by RT-PCR. Sequencing revealed that the N gene amplicons from each sample were 451 bp. RPD rigorous recombination analysis showed a lack of recombination in the N gene region from any of the samples. The N coding region sequences from our 16 patient samples and the 450 downloaded from GenBank were used to create a maximum clade credibility tree (Figure 1). The phylogenetic analysis revealed two clusters represented by the 466 MV N sequences. The first cluster contained genotypes A, B, G, H, and C. The second cluster contained only genotype D, implying that genotype D has a distinctive evolutionary profile. In addition, the 16 newly isolated MV wild-type strains showed the closest relation to genotype H1. This is not surprising since the H1 strain is related to previous outbreaks in China. Based on these results, we defined the epidemic strains of Jilin Province as H1 (indicated in red in Figure 1).

The MV genome-encoded nucleoprotein (N) is critically involved in viral genome transcription and replication, and mediates formation of the MV helical nucleocapsid [13]. The N gene is frequently used for genotyping and phylogenetic analysis of MV. However, no study to date has reported on the relationship between the genetic characteristics of the various MV genotypes and their epidemiologic behavior. Such data would help to identify the source of virus for a particular region or population [14].

### Evolutionary rate and TMRCA of each MV genotype

To date, 23 genotypes of the measles virus have been reported, many of which were identified after and near the year 2000 [17], [18]. To understand the evolutionary behavior of MV both worldwide and in northeastern China, we estimated the dates of origin of each genotype using the Bayesian relaxed molecular clock method. The N gene evolutionary rate was estimated to be 1.127E-3 substitutions/site/year (Table 1). The oldest and youngest genotypes of MV were determined to be D (TMRCA: 45.86) and A (TMRCA: 20.28), respectively. According to our analysis, D and A emerged in 1964.1 and 1989.7, respectively. Table 1 summarizes the times of first report for each genotype included in our analysis. The first report times for genotypes B/D/G/H were several years after the emergence time determined by our analysis. However, the report times for genotypes A/C were far before the time we estimated. We theorize that this inconsistency may be due to the fact that very few sequences of genotypes A/C have been reported, especially during the earliest years of their appearance in the literature.

**Table 1:** Evolutionary characteristics of measles virus genotypes based on the nucleoprotein gene, using the uncorrelated log-normal relaxed clock model, implemented in BEAST.

MV genotype	Location, year reported	HBV TMRCA (years; 95% HPD)	Emergence time
		Substitution rate (CR)* 1.127 (0.925–1.329)	
TMRCA (A)	Northern Ireland, 1956 [19]	20.281 (16.169–26.164)	1989.7
TMRCA (B)	Cameroon, 1983 [20]	26.631 (13.764–41.201)	1983.4
TMRCA (C)	Northern Ireland, 1955 [19]	23.072 (18.161–33.029)	1986.9
TMRCA (D)	Northern Ireland, 1960s	45.862 (31.214–64.844)	1941.1
TMRCA (G)	USA, 1983 [20]	25.371 (16.131–38.058)	1984.6
TMRCA (H)	China, 1993 [21]	34.949 (25.673–46.931)	1975.1
TMRCA (JiLin)	-	6.872 (5.845–7.932)	2003.1

* Substitution rates are expressed as 10^−3^ substitutions per site per year.

The TMRCA for the 16 MV strains from JiLin province was 6.872 (5.845–7.932, 95% CR). Their most recent common ancestor was estimated to have appeared in 2003. This result corresponded to the fact that measles cases in Jilin Province showed a distinct increase in 2005, and reached 18 incidences/100,000 population in 2006 [5]. Considering that viruses usually require a 2–4 year interval to cause epidemic outbreaks [22], the 2005 outbreak of measles in Jilin Province may have in fact been caused by this strain.

### Genetic diversity (g) analysis with the Bayesian skyline plot

**Figure 2:**
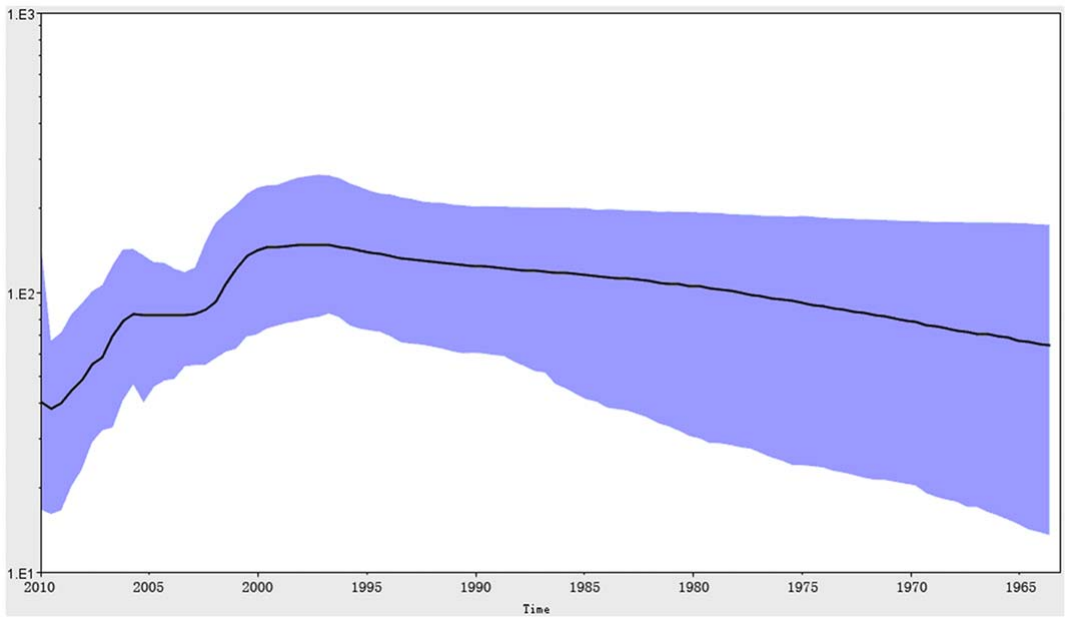
The genetic diversity dynamics of the measles virus N gene estimated by a Bayesian skyline plot through time. The horizontal axis is in units of years, and the vertical axis is Neτ (the product of the effective population size and the generation length in radiocarbon years). The thick solid line is the median estimate, and the dashed lines show the 95% HPD limits. The plot for N gene shows a rise in relative genetic diversity occurring about 10 years ago, in 2000. Very low genetic diversity existed in the early 1970s and in 2010.

We used Bayesian skyline plot analysis [15] to reconstruct the past population history of MV by measuring the dynamics of N gene genetic diversity over time (Figure 2). The highest level of genetic diversity (g) was observed for the N gene in 2009, suggesting that a remarkable amount of MV genetic diversity was occurring at that time. The WHO's statistics data on measles reported cases have indicated that the overall number of MV infection cases in a region is reduced in parallel to improved coverage of measles vaccine immunization. However, in Jilin Province, the number of MV infection cases rose sharply between 1999 and 2001, despite the fact that the region's immunization rate remained high [23]. Furthermore, the incidence of measles in Jilin Province substantially increased in 2009, to about 10 incidences/100,000 population. Again, within those years, the government's immunization program, consisting of routine immunization and supplementary immunization, had continued [5].

According to our Bayesian skyline plot analyses, the MV N gene genetic diversity showed a slight increase from the 1970s to 2000, reaching the highest genetic diversity near 2000. The outbreaks of MV in Jilin Province may be related to the increase in genetic diversity at those times. This phenomenon may also underlie other MV outbreaks across the globe. As the MV genetic diversity in Jilin Province has reduced since 2009, this may be a good time to make a strong increase in China's immunization campaign. Similar permissive periods may exist for other countries, and will be identifiable by the integrated epidemiologic and phylogenetic approach used in our current study. In this way, we may be able to achieve the global target of a 95% reduction in measles mortality from the level seen in 2000 by 2015, as set forth by the World Health Assembly in May 2010.

## Materials and Methods

### Study population

Ethics statement: This study was approved by the independent ethics committee (IEC) of Jilin University. A written informed consent was obtained from the parents after we described the study to them. To evaluate the distribution profile of MV genotypes in Jilin province, 16 MV-positive samples (Table 2) were obtained from 105 patients admitted to our hospital with typical measles symptoms between 2005 and 2006. All 16 samples were stored at −20°C until further use. The geographic distribution of these samples was: Changchun (n = 6), Jilin City (n = 3), SiPing (n = 3), YanBian (n = 1), TongHua (n = 1), and SongYuan (n = 2).

**Table 2:** Summary of natural measles virus strains isolated in Jilin Province, China in 2005–2006.

No.	Age	City	Specimen type
1	20 m	Changchun	Throat swab
2	23 m	Jilin City	Throat swab
3	21 y	Jilin City	Throat swab
4	6 m	Yanbian	Throat swab
5	21 y	Tonghua	Urine
6	27 m	Siping	Throat swab
7	31 m	Siping	Urine
8	21 m	Siping	Urine
9	23 y	Changchun	Throat swab
10	6 m	Songyuan	Throat swab
11	26 m	Changchun	Throat swab
12	29 m	Changchun	Throat swab
13	23 y	Changchun	Throat swab
14	15 y	Songyuan	Throat swab
15	14 y	Changchun	Throat swab
16	20 m	Jilin City	Throat swab

m, months; y, years.

### RNA extraction, reverse transcription-polymerase chain reaction (RT-PCR), and sequencing

The MV samples were amplified in Vero/SLAM (signaling lymphocyte-activation molecule) cells (internal laboratory stock cells) as previously described [24]. Total MV RNA was extracted from the infected cell suspension by using the MiniBEST Viral RNA/DNA Extraction Kit (Ver.4.0; TaKaRa, Shiga, Japan) and following the manufacturer's instructions. The RNA was used as template for RT-PCR amplification of the nucleoprotein (N) gene with the TaKaRa One Step RNA PCR Kit (AMV) and gene-specific primers (forward: 5′-GCT ATG CCA TGG GAG TAG GAG TGG-3′, reverse: 5′-GGC CTC TCG CAC CTA GTC TAG-3′ [25]). RT-PCR products were purified and sent for sequencing at the Beijing Genomics Institute (Shenzhen, China) using a PRISM™ 3730 DNA Sequencer (Applied Biosystems, Inc., Carlsbad, CA, USA).

### Sequence collection and phylogenetic analyses

#### Sequence collection

A total of 476 N gene sequences were downloaded from GenBank (http://www.ncbi.nlm.nih.gov/nucleotide). Of those, 450 had known collection dates (range: 1970–2010), genotypes, and isolate country, and were retrieved for analysis. The accession numbers of these sequences can be found in File S1. These nucleotide sequences were isolated mainly from large measles outbreaks and sporadic cases that occurred globally over the last four decades.

#### Alignment processing and recombination detection

The N sequences were aligned using MEGA software (Ver.5.0 [26]), and edited with the SEAL sequence simulation and alignment evaluation software (http://tree.bio.ed.ac.uk/software/seal/). To perform the phylogenetic analysis, the missing nucleotides were coded as “missing characters” in the nexus block. To test for the presence of recombination in the N gene, sequences were screened using the Recombination Detection Program (RDP3) software package [27]. The highest multiple-comparison-corrected *p*-value cutoff was set at 0.01. In all cases, the best-fit model of nucleotide substitution was determined using the MODELTEST program [28].

#### Bayesian Markov Chain Monte Carlo (MCMC) evolutionary analysis

Bayesian analysis, using the MCMC approach, was performed to construct a maximum clade credibility tree. Convergence was inspected by Tracer (v1.5, http://beast.bio.ed.ac.uk/Tracer), with uncertainties addressed as 95% highest probability density (HPD) intervals. The results were summarized using the TreeAnnotator program with the MCC tree model. Finally, FigTree was used to graphically display the molecular phylogenies. The TMRCA was estimated for six MV genotypes (A–D, G, H) to deduce the oldest and most recently emerged genotypes. The Bayesian skyline plot (BSL) was used to measure the dynamics of N gene genetic diversity over time [15].

The (GTR+G) substitution model was chosen in accordance with the results from the MODELTEST analysis, due to the fact that the generalized time reversible (GTR) approach is considered the most general, neutral, independent approach that accounts for finite-sites and is time-reversible [29]. The “G” value represents the gamma distribution, which is a two-parameter family of continuous probability distributions. Furthermore, the molecular clock model of the Relaxed Clock: Uncorrelated Log-normal was selected for the Bayesian analysis based on the fact that this method assumes no a priori correlation between a lineage's rate of evolution and that of its ancestor. Ten million MCMC runs were sufficient to achieve the convergence of all parameters (effective sampling size >200). Each Bayesian MCMC analysis was run for 20 million states and sampled at every 10,000 states. Posterior probabilities were calculated with a burn-in of 2 million states and checked for convergence using Tracer (v1.5 [30]).

## Supporting Information

File S1 **Access Number and sequences.** A total of 450 N gene sequences were downloaded from GenBank with known collection dates (range: 1970–2010), genotypes, and isolate country, and were retrieved for analysis.
(TXT)

File S2 **Original file of MCC tree build with MV N gene, which can be opened with FigTree v1.3.1 software to be amplified for more details.**
(RAR)
